# HOXC6 predicts invasion and poor survival in hepatocellular carcinoma by driving epithelial-mesenchymal transition

**DOI:** 10.18632/aging.101363

**Published:** 2018-01-15

**Authors:** Pin-Dong Li, Peng Chen, Xin Peng, Charlie Ma, Wen-Jie Zhang, Xiao-Fang Dai

**Affiliations:** 1 Cancer Center, Union Hospital, Tongji Medical College, Huazhong University of Science and Technology, Wuhan 430022, China; 2 Department of Radiation Oncology, Fox Chase Cancer Center, Philadelphia, PA19111, USA; 3 Department of Pathology, Shihezi University School of Medicine, Shihezi, Xinjiang 832002, China

**Keywords:** hepatocellular carcinoma, HOXC6, EMT, aggressiveness, prognosis

## Abstract

Aberrant expression of HOXC6 has been reported in several malignant tumors, yet little is known about the value of HOXC6 in invasion and prognosis of hepatocellular carcinoma (HCC). HOXC6 expression was positively correlated with high AFP level, liver cirrhosis, larger tumor, vascular invasion and BCLC stage. Kaplan-Meier analysis revealed that HOXC6 was an independent predictor for overall survival (OS) and time to recurrence (TTR). In addition, HOXC6 status could act as prognostic predictor in different risk subgroups. Moreover, HOXC6 maintained its prognostic value in different ability of invasiveness. Furthermore, combination of HOXC6 and serum AFP could be a potential predictor for survival in HCC patients. Additionally, further study showed that HOXC6 may promote invasion of HCC by driving epithelial-mesenchymal transition (EMT). Knockdown of HOXC6 significantly decreased the migration and invasion of HCC cells and changed the expression pattern of EMT markers. An opposite expression pattern of EMT markers was observed in HOXC6-transfected cells. In addition, immunohistochemistry and RT-PCR results further confirmed this correlation. In conclusion, HOXC6 contributes to invasion by inducing EMT pathway and predicts poor prognosis of HCC. HOXC6/AFP expression may help to distinguish the different risks of HCC patients after hepatectomy.

## Introduction

Hepatocellular carcinoma (HCC) is the fifth most common cancer worldwide, and its incidence and mortality rates have increased in recent years [1,2]. Although survival of patients with HCC has improved due to advances in surgical techniques and perioperative management, long-term survival after surgical resection remains unsatisfactory because of the high rate of recurrence and metastasis [3,4]. Molecular signatures which define the risk of recurrence and metastatic potential of HCC may allow appropriate therapeutic regimens to be applied earlier in the disease course. Although several prognostic biomarkers in HCC have been reported recently [5,6], there still remains a lack of suitable predictors available that can be widely used in clinical settings [7,8].

Human homeobox (HOX) genes belong to the homeoprotein family of transcription factors, which play critical roles in embryonic development, cell morphogenesis and differentiation regulating numerous processes including apoptosis, receptor signaling, differentiation, motility and angiogenesis [9,10]. In humans, there are 39 HOX genes in four clusters, on chromosomes 7, 17, 12 and 2 for the HOXA, HOXB, HOXC and HOXD clusters, respectively [11]. Many HOX genes are involved in tumor cell proliferation, metastasis and angiogenesis, and levels of these genes are correlated with patients’ outcome [12]. Therefore, HOX genes play an important role in a variety of cancers, and are potential predictors for disease diagnosis, and target for novel therapies.

Homeobox C6 (HOXC6) that is localized on chromosomes 12q13.3 shares a 5’ non-coding exon with HOXC5 and HOXC4 [13,14]. Previous studies showed that HOXC6 could be regulated by MLL2, MLL3, TGF and hormones [15–18]. HOXC6 regulates genes with both oncogenic and tumor suppressor activities through the regulation of its functional biological targets bone morphogenic protein 7, platelet-derived growth factor receptor and fibroblast growth factor receptor 2, as well as PI3K/AKT, Notch and Wnt pathways [19–22]. Overexpression of HOXC6 has been reported in breast [17], lung [14], gastric [23], colorectal [24], and prostate carcinomas [25]. In addition, it also reported that HOXC6 could inhibit the proliferation, migration and chemosensitivity of HCC cells *in vitro* [26]. However, the role and mechanism of HOXC6 in prognosis of HCC patients has not been well clarified.

In present study, we detected the expression of HOXC6 in HCC samples and HCC cells, and evaluated clinicopathological parameters significance and prognosis of HOXC6 expression in HCC patients. Epithelial-mesenchymal transition (EMT) is a key process in cancer metastasis by which tumor cells acquire migratory characteristics, thereby disassociating from the primary tumor and migrating to distant sites. We therefore determined correlation of HOXC6 expression with EMT markers *in vitro*. Furthermore, serum α-fetoprotein (AFP) level is a poor prognostic marker for HCC patients [27]. Therefore, we also investigated the prognosis of HOXC6 combined with serum AFP level in HCC patients.

## RESULTS

### Relationship between HOXC6 and clinicopathological features

**Figure 1:**
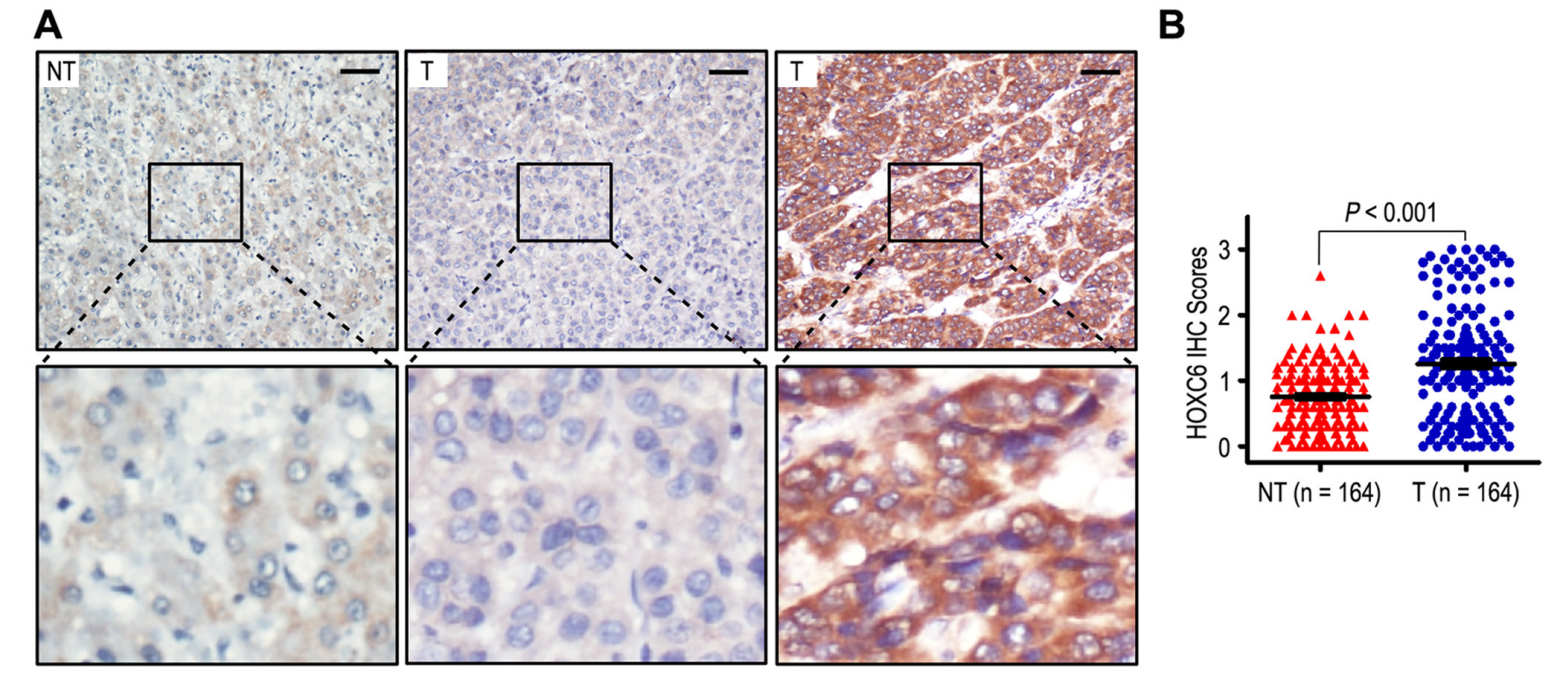
**HOXC6 was overexpression in hepatocellular carcinoma samples.** (**A**) Immunohistochemistry assays of HOXC6 expression in HCC tissues and adjacent non-tumorous tissues. The upper left panel represents low HOXC6 expression in adjacent non-tumorous tissues. The upper middle and right panel represents low and high HOXC6 expression in HCC tissues. Lower panels represent magnified pictures of boxed area in the corresponding upper panels. The line scale bar represents 50 μm. (**B**) HOXC6 expression in HCC was compared with that in adjacent non-tumorous specimens. Statistical analysis was performed by Paired-Samples *t*-test.

In order to analyze the importance of HOXC6 in HCC patients, we detected the expression of HOXC6 in 164 HCC samples and matched adjacent non-tumorous liver tissues by immunohistochemistry. Representative images indicated that HOXC6 was mainly localized in cytoplasm of HCC cells (Fig. 1A). A total of 88 samples exhibited high expression, and 76 exhibited low expression. The expression of HOXC6 in tumors is significantly higher than that in adjacent non-tumorous tissues (*P* < 0.001; Fig. 1B), suggesting that HOXC6 accumulated in HCC samples.

To investigate the biological functions of HOXC6 in HCC patients, we determined the correlation of HOXC6 status with clinicopathological features in 164 HCC tissues. HOXC6 overexpression correlated with high AFP level (*P* = 0.038), liver cirrhosis (*P* = 0.044), larger tumor (*P* = 0.001), vascular invasion (*P* = 0.046) and Barcelona Clinic Liver Cancer (BCLC) stage (*P* = 0.005) (Table S1). On the contrary, HOXC6 expression had no correlation with other features, such as gender, age, HBsAg, gamma-glutamyltransferase (GGT), tumor number, satellite nodule, tumor margin, tumor differentiation and tumor-node-metastasis (TNM) stage (all *P* > 0.05). In addition, HOXC6 expression in tumors gradually increased in TNM and BCLC stage-dependent manner (Fig. S1).

### HOXC6 overexpression in tumors indicated poor prognosis

To determine the significance of HOXC6 status on prognosis in HCC patients, we analyzed univariate analysis of clinicopathologic characteristics and HOXC6 expression for Overall survival (OS) and time to recurrence (TTR). Kaplan-Meier analysis showed that the patients with high expression of HOXC6, high AFP level, high gamma-glutamyltransferase (GGT) level, liver cirrhosis, larger tumor or vascular invasion had poorer OS time (all *P* < 0.05). Furthermore, high HOXC6 expression, high AFP level, high GGT level, liver cirrhosis, larger tumor, tumor number, satellite nodule and vascular invasion were unfavorable predictors for TTR of HCC patients (all *P* < 0.05, Table 1).

**Table 1:** Univariate and multivariate analysis of HOXC6 associated with survival and recurrence in HCC patients.

Variables*	OS				TTR			
	Univariate	Multivariate			Univariate	Multivariate		
	*P*-value	*P*-value	HR	95% CI	*P*-value	*P*-value	HR	95% CI
Gender (Female vs. Male)	NS	NS			NS	NS		
Age, years (> 50 vs. ≤ 50)	NS	NS			NS	NS		
AFP (ng/mL) (> 400 vs. ≤ 400)	< 0.001	0.004	2.081	1.271-3.407	0.001	0.014	1.805	1.129-2.886
HBsAg (Positive vs. Negative)	NS	NS			NS	NS		
GGT (U/l) (> 50 vs. ≤ 50)	0.017	NS			0.031	NS		
Liver cirrhosis (Yes vs. No)	0.021	0.050	2.067	0.999-4.275	0.041	NS		
Tumor size (cm) (> 5 vs. ≤ 5)	< 0.001	NS			< 0.001	0.019	1.875	1.110-3.169
Tumor number (Multiple vs. Single)	NS	NS			0.023	0.042	2.191	1.028-4.669
Tumor margin (≥ 2 vs. < 2)	NS	NS			NS	NS		
Satellite nodule (Yes vs. No)	NS	NS			0.014	NS		
Tumor differentiation (III-IV vs. I-II)	NS	NS			NS	NS		
Vascular invasion (Yes vs. No)	< 0.001	< 0.001	3.139	1.760-5.601	< 0.001	< 0.001	3.006	1.727-5.230
HOXC6 (High vs. Low)	< 0.001	0.001	2.543	1.437-4.498	< 0.001	< 0.001	3.540	2.047-6.122

*TNM stage and BCLC stage was combined with several clinical indexes such as tumor size, number and tumor thrombus; we did not enter the TNM stage and BCLC stage into multiple analysis with these indexes to avoid any bias in analysis.

GGT gamma-glutamyltransferase, AFP α-fetoprotein, OS overall survival, TTR time to recurrence, NS not significant, HR hazard ratio, CI confidential interval.

**Figure 2:**
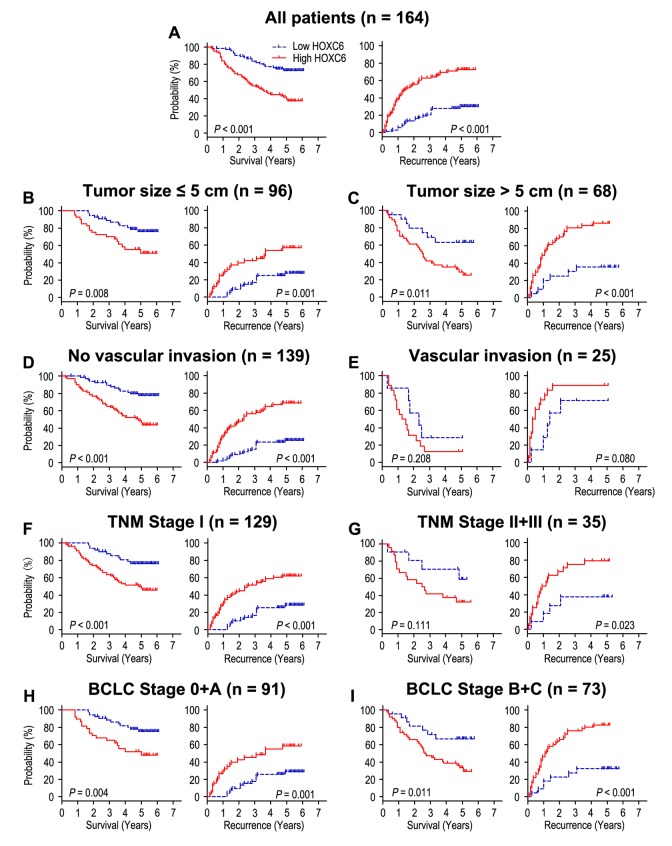
**Overall survival and time to recurrence are shown for patients with HCC.** All patients were classified according to tumor size, vascular invasion, TNM stage and BCLC stage. Kaplan-Meier survival estimates and log-rank tests were used to analyze the prognostic value of HOXC6 expression in all patients (**A**) and each subgroup (**B**-**I**).

Significant OS and TTR advantages were observed in patients with HOXC6 low-expression (both *P* < 0.001) (Fig. 2A). Moreover, the median of OS and TTR times in HOXC6 overexpression group (n = 88) were 38.5 months and 17.5 months, while there were 58.0 months and 58.0 months in HOXC6 low expression group (n = 76). In addition, the 5-year OS and TTR rates of the HOXC6 overexpression group were significantly lower than those of the HOXC6 low expression group (OS: 41.5% *vs*. 72.8%, TTR: 27.4% *vs*. 70.2%) (Fig. 2A). Moreover, multivariate analysis was used to analyze whether HOXC6 could be an independent prognostic marker for OS and TTR in HCC patients. Our results found that overexpression of HOXC6 remained as an independent predictor in multivariate models and portended worse OS in HCC patients (HR = 2.543, 95% CI = 1.437-4.498, *P* = 0.001). The patients with HOXC6 overexpression will be more likely to suffer from recurrence than those with low HOXC6 expression (HR = 3.540, 95% CI = 2.047-6.122, *P* < 0.001), (Table 1).

To discuss prognostic significance of HOXC6 in different subgroups, HCC patients were divided base on tumor size (Fig. 2B, C), vascular invasion (Fig. 2D, E), TNM stage (Fig. 2F, G) and BCLC stage (Fig. 2H, I). Patients with HOXC6 overexpression predicted unfavourable OS and TTR times in all of these subgroups for except OS and TTR in patients who had vascular invasion (*P* = 0.208, and *P* = 0.080) or OS in patients with TNM stage II/III (*P* = 0.111). In general, it reveals that HOXC6 status could act as prognostic predictor in different risk subgroups of HCC patients.

### HOXC6 overexpression predicts unfavorable prognosis independent of tumor invasiveness

**Figure 3:**
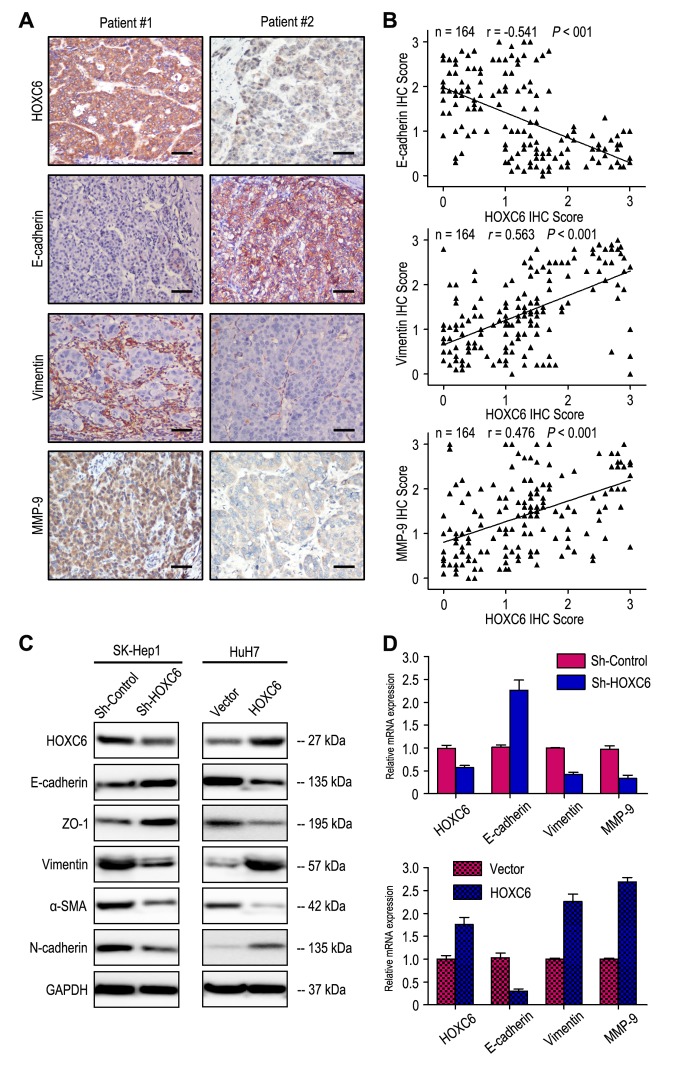
**HOXC6 expression level correlated with the expression of epithelial-mesenchymal transition (EMT) markers.** (**A**) Serial sections of human HCC tissue were subjected to IHC staining with antibodies against HOXC6, E-cadherin, Vimentin and MMP-9. In patient #1, high expression of HOXC6 in HCC tissues was accompanied by the absence of E-cadherin and elevated Vimentin, MMP-9. In patient #2, low expression of HOXC6 was accompanied by elevated E-cadherin and the absence of Vimentin, MMP-9. The scale bar represents 50 μm. (**B**) HOXC6 expression was negatively correlated with E-cadherin expression and positively associated with vimentin and MMP-9 expression. (**C**) Decreased expression of Vimentin, α-SMA and N-cadherin with increased expression of E-cadherin and ZO-1 in HOXC6-silenced cells compared with the control cells. An opposite expression pattern of these genes was observed in HOXC6-transfected cells. (**D**) The mRNA of E-cadherin was up-regulated, while the vimentin and MMP-9 was down-regulated, when HOXC6 was silenced. And opposite expression was observed in HOXC6-transfected cells.

**Figure 4:**
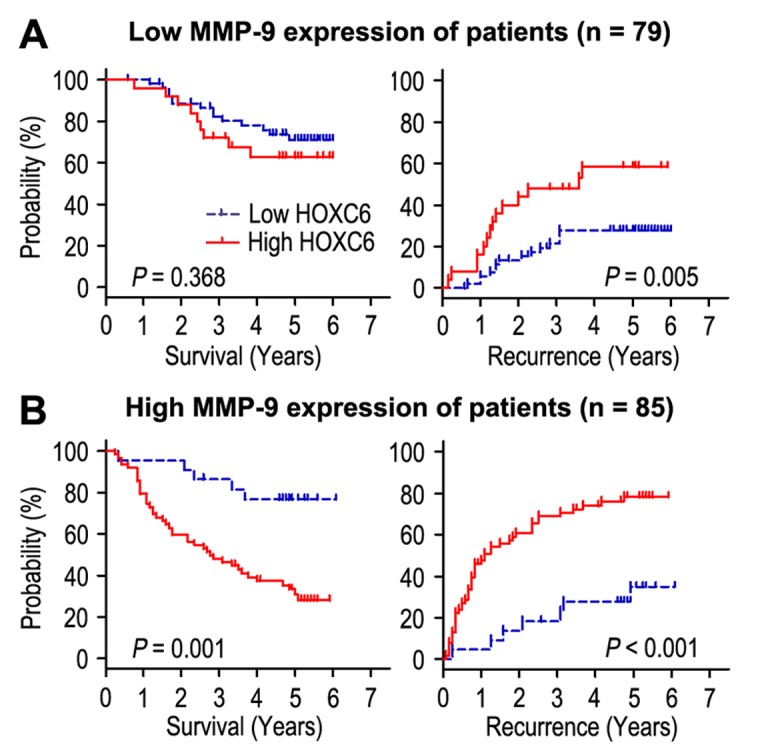
**Overall survival and time to recurrence are shown for patients with low MMP-9 expression** (**A**) and high MMP-9 expression (**B**). Kaplan-Meier survival estimates and log-rank tests were used to assess the association between HOXC6 expression and overall survival or time to recurrence in patients with low MMP-9 expression (n = 79) or high MMP-9 (n = 85).

The correlation of HOXC6 and matrix metallopeptidase 9 (MMP-9) expression was evaluated by immunohistochemistry (IHC) assays in serial sections of HCC samples (Fig. 3A). Our results revealed that HOXC6 expression had positively correlation with MMP-9 expression in HCC tissues (*r* = 0.476, *P* < 0.001, Fig. 3B) and HCC cells (Fig. 3D). We further investigated the effect of HOXC6 expression on prognosis independent of tumor invasiveness by using MMP-9 marker as an indicator for invasive potential of tumor cells. The HCC patients were stratified into low invasiveness group (low MMP-9 expression; n = 79) and high invasiveness group (high MMP-9 expression; n = 85). In patients with low invasiveness subgroup, HOXC6 overexpression was associated with worse TTR (*P* = 0.005), while there was no significance in OS time (Fig. 4A). In the high tumor invasiveness group (Fig. 4B), patients with high HOXC6 expression were likely to death (*P* = 0.001) and recurrence (*P* < 0.001) compared with those with low HOXC6 expression. Therefore, HOXC6 status may be a prognostic predictor for HCC patients independent of tumor invasiveness.

### Combined influence of HOXC6 and serum AFP on risk of HCC prognosis

**Figure 5:**
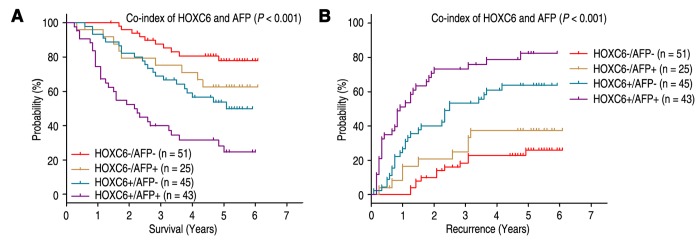
**Combined value of HOXC6 and serum AFP identify different risks of HCC death and recurrence.** The associations of HOXC6/AFP co-expression with Overall survival (log-rank *P* < 0.001) (**A**) and Time to recurrence (log-rank *P* < 0.001) (**B**) in 164 HCC patients.

Previous study revealed that high serum AFP level was associated with poor prognosis for HCC patients [27]. Prognostic analysis showed patients with preoperative serum AFP level above 400 ng/mL had worse OS (*P* < 0.001) and shorter TTR (*P* = 0.001), and could be an independent predictor in HCC patients (Table 1). Furthermore, we combined the prognostic significance of HOXC6 expression with serum AFP levels in HCC patients. According to the expression of HOXC6 and serum AFP levels, the prognosis of patients were classified into four different risk subgroups: patients with HOXC6 (-) and AFP ≤ 400 ng/ml (HOXC6-/AFP-) had better OS and TTR time; patients with HOXC6 (-) and AFP > 400 ng/ml (HOXC6-/AFP+), or patients with HOXC6 (+) and AFP ≤ 400 ng/ml (HOXC6+/AFP-), had intermediate prognosis and intermediate-risk of relapse; patients with HOXC6 (+) and AFP > 400 ng/ml (HOXC6+/AFP+) were prone to death and relapse (Fig. 5). Multivariate analysis showed that co-index of HOXC6/AFP could be used as an independent prognostic marker for OS (HR = 1.650, 95% CI = 1.307-2.083, *P* < 0.001) and TTR (HR = 1.841, 95% CI = 1.478-2.292, *P* < 0.001) (Table 2).

**Table 2:** Univariate and multivariate analysis of co-index of HOXC6/AFP associated with survival and recurrence in HCC patients.

Variables	OS				TTR			
	Univariate	Multivariate			Univariate	Multivariate		
	*P*-value	*P*-value	HR	95% CI	*P*-value	*P*-value	HR	95% CI
Gender (Female vs. Male)	NS	NS			NS	NS		
Age, years (> 50 vs. ≤ 50)	NS	NS			NS	NS		
HBsAg (Positive vs. Negative)	NS	NS			NS	NS		
GGT (U/l) (> 50 vs. ≤ 50)	0.017	NS			0.031	NS		
Liver cirrhosis (Yes vs. No)	0.021	NS			0.041	NS		
Tumor size (cm) (> 5 vs. ≤ 5)	< 0.001	NS			< 0.001	0.030	1.783	1.058-3.004
Tumor number (Multiple vs. Single)	NS	NS			0.023	0.004	3.085	1.438-6.617
Tumor margin (≥ 2 vs. < 2)	NS	NS			NS	NS		
Satellite nodule (Yes vs. No)	NS	NS			0.014	NS		
Tumor differentiation (III-IV vs. I-II)	NS	NS			NS	NS		
Vascular invasion (Yes vs. No)	< 0.001	< 0.001	3.232	1.819-5.745	< 0.001	< 0.001	3.225	1.856-5.605
Co-index of HOXC6/AFP*	< 0.001	< 0.001	1.650	1.307-2.083	< 0.001	< 0.001	1.841	1.478-2.292

*Co-index of HOXC6/AFP was combined with HOXC6 and AFP, therefore, we did not enter the HOXC6 and AFP into univariate and multiple analysis with these indexes to avoid any bias in analysis.

GGT gamma-glutamyltransferase, AFP α-fetoprotein, OS overall survival, TTR time to recurrence, NS not significant, HR hazard ratio, CI confidential interval.

### HOXC6 expression promoted HCC cell invasion via EMT pathway

**Figure 6:**
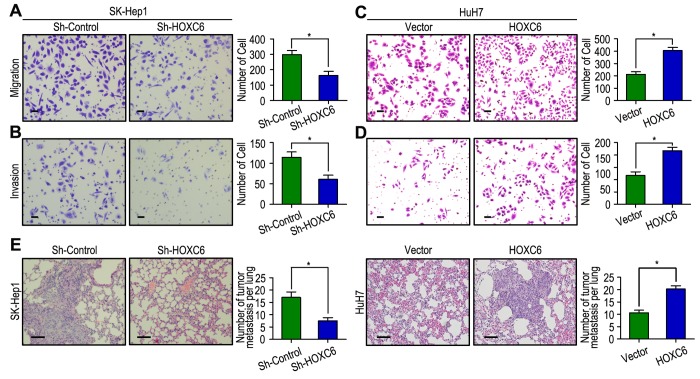
**HOXC6 promotes cell migration and invasion in HCC cell lines.** (**A**, **B**) HOXC6 silenced in SK-Hep1 cells and inhibited cell migration and invasion. (**C**, **D**) HOXC6 up-regulated in HuH7 cells and promoted cell migration and invasion. (**E**) Lung H&E staining of nude mice inoculated SK-Hep1 and HuH7 transfected cells via tail vein. The number of lung metastatic foci in each group were also calculated, * *P* < 0.05.

We further determined the significance of HOXC6 in tumor cell migration and invasion, which has been implicated from clinical data. SK-Hep1 cells migrated slower and had less ability to invade through Matrigel when HOXC6 was knocked down (Fig. 6A and B). In contrast, HuH7 cells migrated faster and had more invasive ability when HOXC6 was upregulated (Fig. 6C and D). We further evaluated the effect of HOXC6 on tumor metastasis in SK-Hep1 and HuH7 xenografts *in vivo*. After 8 weeks, the mice were sacrificed, and the metastatic nodules at the lung surfaces were counted. There were less numbers of metastatic nodules at the surface of the lungs of mice when HOXC6 was silenced. In contrast, with the HuH7 cells, tumors developed from HOXC6-transfected cells were significantly larger than tumors from vector cells. Hematoxyliin and eosin (H&E) staining confirmed that the nodules on the surfaces of mice lungs were metastatic tumors (Fig. 6E). These data suggest that enhanced HOXC6 may promote the motile and invasive abilities of HCC cells.

As EMT is one of the key events in tumor invasion and metastasis, the effect of HOXC6 on EMT markers was analyzed. The expression of HOXC6 was negatively correlated with E-cadherin expression and positively associated with Vimentin and MMP-9 expression in HCC samples (Fig. 3A and B). Interestingly, our western blot results found that decreased expression of Vimentin, α-SMA and N-cadherin with increased expression of E-cadherin and ZO-1 in HOXC6-silenced cells compared with the control cells. An opposite expression pattern of these genes was observed in HOXC6-transfected cells (Fig. 3C). Similar to the results of western blotting, our RT-PCR results showed that up-regulation of E-cadherin and down-regulation of Vimentin and MMP-9 when HOXC6 was silenced, while opposite expression was observed in HOXC6-transfected cells (Fig. 3D). Together, our results suggest that HOXC6 may promote HCC cells invasion by driving EMT pathway.

## DISCUSSION

HOXC6 is crucial to cell development and proliferation in response to hormonal signals. In addition, HOXC6 deregulation has been detected in several cancer types. Although HOXC6 is reported to promote cell proliferation and migration in HCC [26], the prognosis value of HOXC6 in HCC patients remains unclear. In this study, we found that the expression of HOXC6 in tumor was higher than that in adjacent non-tumorous tissues. In addition, HOXC6 overexpression was positively associated with high AFP level, liver cirrhosis, larger tumor, vascular invasion and BCLC stage. The expression levels of HOXC6 were significantly increased in TNM and BCLC stage-dependent manner. Sui CJ et al. [26] reported siRNA-mediated HOXC6 downregulation was able to inhibit cell proliferation and migration in HepG2 and HuH7 cells. These results suggested that HOXC6 expression may be correlated with the progression of HCC. Moreover, the Kaplan-Meier curves revealed that patients with high HOXC6 expression had lower OS and TTR rates than did the group with low HOXC6 expression. Multivariate Cox regression analysis of the HCC patients showed that HOXC6 overexpression was an independent prognostic factor for HCC patients. Moreover, the prognostic significance of HOXC6 in different risk of subgroups according to tumor size, vascular invasion, TNM stage and BCLC stage was also assessed, which showed that HOXC6 could maintain its prognostic value in different risk of HCC patients. Our findings had similar results with previous study. Sui CJ et al. [26] found that siRNA mediated HOXC6 knockdown inhibited *in vitro* proliferation and migration, and increased 5-FU chemosensitivity in HCC cell. On the other hand, HOXC6 overexpression reversed the inhibitory effect of miR-147 on HCC *in vitro* proliferation. Zhang Q et al. [23] reported that HOXC6 mRNA was increased in gastric cancer tissues compared with that in the adjacent normal mucosa. The expression of HOXC6 was associated with depth of invasion. Moreover, patients with high HOXC6 expression had shorter overall survival rate. Ji ML et al. [24] also revealed that HOXC6 was more abundantly expressed in colorectal cancer (CRC) and was an independent risk factor for poor CRC patients prognosis. HOXC6 may promote CRC cell proliferation and tumorigenesis through autophagy inhibition and mTOR pathway activation. Our results and previous foundings imply that high HOXC6 expression is associated with tumor progression and may act as potential prognostic biomarker for HCC patients.

It has been known that degradation of extracellular matrix (ECM) could be the signal for the beginning of invasion and metastasis in tumor, and matrix metalloproteases play an important role in degradation of ECM during invasion and metastasis [28]. Previous study showed that MMP-9 gene regulated the bioavailability of growth factors and to disrupt cell-cell contacts, dramatically impacting cell proliferation, migration and survival [29]. Arii S et al. [30] found that the expression of MMP-9 mRNA in HCC with capsular infiltration was high compared with that in HCC without capsular infiltration, which suggest that MMP-9 is closely participated in capsular infiltration in HCC. Moreover, MMP-9 immunoreactivity was more intense in the HCC cells, particularly in those cells in the marginal areas of the tumorous tissues. MMP-9 overexpression is an independent poor prognostic biomarker for HCC patients [31]. In our study, the expression of HOXC6 was closely correlated with MMP-9 expression in HCC samples, suggesting that the tumor cells with high HOXC6 expression had high invasiveness. Furthermore, HOXC6 overexpression was a factor for poor prognosis in patients with HCC independent of tumor cell invasiveness. In general, HOXC6 status in HCC accelerating tumor progression reveals that HOXC6 may be a promising target in cancer therapy.

AFP is a frequently-used tumor biomarkers for the diagnosis and predicting prognosis of HCC, and monitoring metastasis and relapse in HCC patients with high AFP level after hepatectomy [32,33]. However, it is difficult to predict the survival and metastatic relapse for HCC patients who have normal AFP level after liver resection. To discuss whether the prognostic significance of HOXC6 expression combined with serum AFP level was superior to AFP alone, the HCC patients were stratified into four subgroups on the basis of HOXC6 expression and serum AFP level. Our results revealed that co-index of HOXC6 expression and serum AFP level could be more beneficial for differentiating the risk of tumor survival and relapse of patients than HOXC6 expression or serum AFP alone. Therefore, the simultaneous analysis of HOXC6 expression and serum AFP level may help determine whether adjuvant therapy is required after resection.

Our functional studies found that HOXC6 had strong tumorigenicity, with overexpression of HOXC6 promoting tumor cell migration and invasion. These effects were shown to be effectively inhibited when the HOXC6 gene was silenced with shRNAs. The role of HOXC6 in promoting tumor metastasis was further supported by the *in vivo* experimental metastasis assay. Because that EMT play an important role in tumor invasion and metastasis, and EMT can increase the invasion of cancer cells [34], we further detected whether the effect of HOXC6 on cell motility was via induction of the EMT pathway. As expected, HOXC6 expression was negatively correlated with E-cadherin, ZO-1 and positively correlated with vimentin, α-SMA, N-cadherin. Moreover, the relationship of HOXC6 and EMT markers, E-cadherin and vimentin, was confirmed in tissue samples and HCC cells. Therefore, HOXC6 may enhance HCC invasion by inducing EMT, thereby contributing to poor prognosis.

## CONCLUSIONS

In summary, our results showed that HOXC6 may promote tumor progression by inducing EMT pathway and can serve as a feasible prognostic predictor for HCC patients. In addition, the co-index of HOXC6 expression with serum AFP level may help to distinguish the different risks of HCC patients after hepatectomy. The identification of HCC patients who are at a high risk of early relapse may provide clinicians with opportunities for early interventions and improve the survival of HCC. The findings of the current study may help to determine personalized adjuvant therapies for HCC patients. However, the mechanism by which HOXC6 involved in tumorigenesis and cancer progression has not been elucidated yet.

## MATERIALS AND METHODS

### Patients and specimens

The research was approved by the Institutional Review Board and Human Ethics Committee of Cancer Center, Union Hospital, Tongji Medical College, Huazhong University of Science and Technology. Written informed consent was obtained from all the patients enrolled in this study.

All HCC samples were obtained from 164 consecutive patients who underwent hepatectomy from February 2009 to December 2010 at the Cancer Center, Union Hospital, Tongji Medical College, Huazhong University of Science and Technology (Wuhan, China). The inclusion criteria of this study was as follows: Firstly, all the cases were tested for pathological proof. Secondly, none of the patients had distant metastasis or received previous therapy before liver resection. Finally, there were no serious complications in perioperative period or no other malignant tumor. All the patients were chosen consistently according to availability of resection samples and follow-up data. The clinicopathological characteristics of HCC patients were showed in (Table S2). Tumor stage was determined based on the 7th Edition tumor-node-metastasis (TNM) classification of the American Joint Committee on Cancer Staging and the Barcelona Clinic Liver Cancer (BCLC) staging system. Overall survival was defined from date of operation to date of death or last follow-up. Time to recurrence was computed as from date of operation to date of recurrence or last follow-up.

### Cell culture

Two human HCC cell lines were used in this study: SK-Hep1 and HuH7. The two cell lines were obtained from Cell Bank of Shanghai Institutes for Biological Sciences, Chinese Academy of Sciences (Shanghai, China). SK-Hep1 and HuH7 were maintained in DMEM supplemented with 10% fetal bovine serum and 100 units/ml penicillinstreptomycin (Invitrogen, USA) at 37°C in 5% CO_2_.

### IHC staining

All 164 HCC tissues and their para-carcinoma samples were detected by IHC method. Slides were baked at 55 °C for 1.5 h, deparaffinized with xylene and rehydrated using an alcohol gradient (100% alcohol, 95% alcohol, 80% alcohol, and 70% alcohol). The tissue slides were then treated with 3% hydrogen peroxide in methanol for 30 min to quench endogenous peroxidase activity, and the antigens were retrieved in 0.01 M sodium citrate buffer (pH 6.0) using a microwave oven. After antigen retrieval, the sections were incubated with 10% normal goat serum to block any nonspecific reaction, the sections were incubated using a primary antibody against HOXC6 (Anti-HOXC6 antibody produced in mouse, sc-376330, Santa Cruz Biotechnology, USA) or E-cadherin (Anti-E-cadherin antibody produced in mouse, Abcam, UK) or Vimentin (Anti-Vimentin antibody produced in rabbit, Abcam, UK) or MMP-9 (Anti-MMP-9 antibody produced in rabbit, Abcam, UK), at 4 °C overnight. The tissue slides were treated with a non-biotin horseradish-peroxidase detection system according to the manufacturer’s instructions (Gene Tech). Immunohistochemical signals were scored by two independent investigators blinded to the patients’ identity and clinical status. In discrepant cases, a pathologist reviewed the cases, and a consensus was reached.

Both the extent and intensity of immunohistochemical staining were taken into consideration when analyzing the data. The intensity of staining was scored from 0 to 3, and the extent of staining was scored from 0% to 100%. The final quantitation of each staining was obtained by multiplying the two scores. HOXC6 expression was classified as high expression if the score was higher than the median score of 1.1, if the score was 1.1 or less, the case was classified as low expression.

### Plasmid constructs and transfection

cDNA containing open reading frames of HOXC6 was amplified by PCR and cloned into pcDNA3.1 vector (Invitrogen, Carlsbad, CA), and then transfected into HuH7 cell using Lipofectamine 2000 (Invitrogen) according to the manufacturer’s instructions. Cell transfected with empty vector was used as control. Stable HOXC6-expressing clones were selected by Geneticin (Rache Diagnostics, Indianapolis, IN) at the concentration of 500ug/ml.

### Establishment of HOXC6 knockdown cells

Short hairpin RNAs (shRNA) targeting the human HOXC6 gene was purchased from GeneRay Biotechnology (GeneRay Biotechnology Co., Ltd.) and transfected into SK-Hep1 cell using Lipofectamine 2000 (Invitrogen) base on the manufacturer’s guidelines. Cell transfected with empty vector was used as control. Two days after transfection, cell culture media was collected and concentrated. Real-time PCR and Western blot were used to validate gene silencing efficiency of shRNA plasmids 48 hours after transfection. The recombinant lentivirus was stored at -80°C.

### Total RNA extraction and qRT-PCR

Total cellular RNA was extracted from HCC cell lines using TRIzol reagent (Life Technologies, USA) base on the manufacturer’s instructions. RNA (2 µg) was reverse transcribed using a PrimeScript RT Kit (Takara, Japan). Real-time PCR was performed using SYBR master mix (Takara) on the Bio-Rad Connect Real-Time PCR platform. The primer sequences used for human HOXC6 were 5’-CACCGCCTATGATCCAGTGAGGCA-3’ (forward) and 5’-GCTGGAACTGAACACGACATTCTC-3’ (reverse). Relative mRNA quantities were determined using comparative cycle threshold methods and normalized against GAPDH.

### Western blotting

Western blot analyses were performed according to the standard protocol. The primary antibodies used for western blot were described in IHC method.

### Transwell invasion assays

Invasion assay was performed with BD BioCoat Matrigel Invasion Chambers (Becton Dickinson Labware, Franklin Lakes, NJ) following the manufacturer’s instructions. The matrigel membrane was stained with crystal violet, and migrated cells were counted under a microscope.

### Experimental metastasis assay

All animal experiments were performed in accordance with NIH guidelines for the use of experimental animals. Male athymic BALB/c nude mice were purchased from the Shanghai Experimental Animal Center of Chinese Academic of Sciences (Shanghai, China). Two groups of 8 mice each were given intravenous tail vein injections of 1×10^6^ shControl-SK-Hep1 cells and shHOXC6-SK-Hep1 cells, or 2×10^6^ vector-HuH7 cells and HOXC6-HuH7 cells, respectively. After 8 weeks, the mice were sacrificed, and the tumor nodules formed on the lung surfaces were counted. Lungs were excised and embedded in paraffin for further study.

### Follow-up

The last follow-up date was on 30 March 2017. In all the HCC patients (21 females and 143 males), the median follow-up period was 52.5 months (ranging 3.0 - 73.0 months). All patients were conducted once in every 3 months in the first 2 year, once in every 6 months from 2 to 5 year. The follow-up protocol included physical examination, serum AFP level and abdominal ultrasonography. Computed tomography and/or magnetic resonance imaging and/or positron emission tomography were performed when intrahepatic relapse or distant metastasis was suspected. During the follow-up period, there were 83 (50.6%) patients who suffered relapse and 70 patients (42.7%) died of cancer-related causes. Ninety-four patients were still alive at the time of the last follow-up report.

### Statistical analysis

Statistical analysis was performed using SPSS 16.0 statistical software (SPSS Inc, Chicago, IL). The relationship between the expression of HOXC6 and clinicopathologic characteristics was tested by χ2 test. The Student’s *t*-test was used for comparisons. Pearson χ2 test was applied to analyze the correlation of HOXC6 with EMT markers. Multivariate analysis was performed using the Cox proportional hazards model. Kaplan-Meier analysis and log-rank tests were also performed to compare prognostic differences between two groups. *P* values < 0.05 were considered statistically significant.

## Supplementary Material

Supplementary File
