# Supplementary material for: HOXC6 predicts invasion and poor survival in hepatocellular carcinoma by driving epithelial-mesenchymal transition
Source: Aging (Albany NY). 2018 Jan 15;10(1):115–30. doi: 10.18632/aging.101363 (PMC5811246; doi:10.18632/aging.101363)
Supplement: Supplementary File [file aging-10-101363-s001.pdf]

SUPPLEMENTARY MATERIAL

Table S1. Correlation of HOXC6 protein expression with clinicopathological parameters.

Characteristics	No. of patients	HOXC6 expression (%)		*P*-value
		Low	High	
Gender				
Female	21	10 (47.6%)	11 (52.4%)	0.900
Male	143	66 (46.2%)	77 (53.8%)	
Age (years)				
≤ 50	89	36 (40.4%)	53 (59.6%)	0.099
> 50	75	40 (53.3%)	35 (46.7%)	
AFP (ng/ml)				
≤ 400	96	51 (53.1%)	45 (46.9%)	0.038
> 400	68	25 (36.8%)	43 (63.2%)	
HBsAg				
Negative	11	5 (45.5%)	6 (54.5%)	0.951
Positive	153	71 (46.4%)	82 (53.6%)	
GGT (U/l)				
≤ 50	90	47 (52.2%)	43 (47.8%)	0.096
> 50	74	29 (39.2%)	45 (60.8%)	
Liver cirrhosis				
No	36	22 (61.1%)	14 (38.9%)	0.044
Yes	128	54 (42.2%)	74 (57.8%)	
Tumor size (cm)				
≤ 5	96	55 (57.3%)	41 (42.7%)	0.001
> 5	68	21 (30.9%)	47 (69.1%)	
Tumor number				
Single	151	71 (47.0%)	80 (53.0%)	0.553
Multiple	13	5 (38.5%)	8 (61.5%)	
Satellite nodule				
No	142	70 (49.3%)	72 (50.7%)	0.054
Yes	22	6 (27.3%)	16 (72.7%)	
Tumor margin				
< 2	102	48 (47.1%)	54 (52.9%)	0.813
≥ 2	62	28 (45.2%)	34 (54.8%)	
Tumor differentiation				
I-II	116	54 (46.6%)	62 (53.4%)	0.933
III-IV	48	22 (45.8%)	26 (54.2%)	
Vascular invasion				
No	139	69 (49.6%)	70 (50.4%)	0.046
Yes	25	7 (28.0%)	18 (72.0%)	
TNM stage				
I	129	65 (50.4%)	64 (49.6%)	0.067
II	8	4 (50.0%)	4 (50.0%)	
III	27	7 (25.9%)	20 (74.1%)	
BCLC stage				
0	17	9 (52.9%)	8 (47.1%)	**0.005**
A	74	44 (59.5%)	30 (40.5%)	
B	58	20 (34.5%)	38 (65.5%)	
C	15	3 (20.0%)	12 (80.0%)	

Table S2. Patient characteristics.

Variable	No. of patients (%)
No. of patients	164 (100)
Age: Median [range], y	46 [13-77]
Gender	
Female	21 (12.8)
Male	143 (87.2)
HBsAg	
Negative	11 (6.7)
Positive	153 (93.3)
AFP: Median [range], ng/mL	142.4 [0.6-121000]
GGT: Median [range], U/l	42.6 [4.5-610.5]
Tumor size: Median [range], cm	4.0 [1.0-18.0]
Liver cirrhosis	
No	36 (22.0)
Yes	128 (78.0)
Tumor number	
Single	151 (92.1)
Multiple	13 (7.9)
Tumor margin	
< 2	102 (62.2)
≥ 2	62 (37.8)
Satellite nodule	
No	142 (86.6)
Yes	22 (13.4)
Tumor differentiation	
I	7 (4.3)
II	109 (66.5)
III	42 (25.6)
IV	6 (3.6)
Vascular invasion	
No	139 (87.8)
Yes	25 (12.2)
TNM stage	
I	129 (78.7)
II	8 (4.9)
III	27 (16.4)
BCLC stage	
0	17 (10.4)
A	74 (45.1)
B	58 (35.4)
C	15 (9.1)

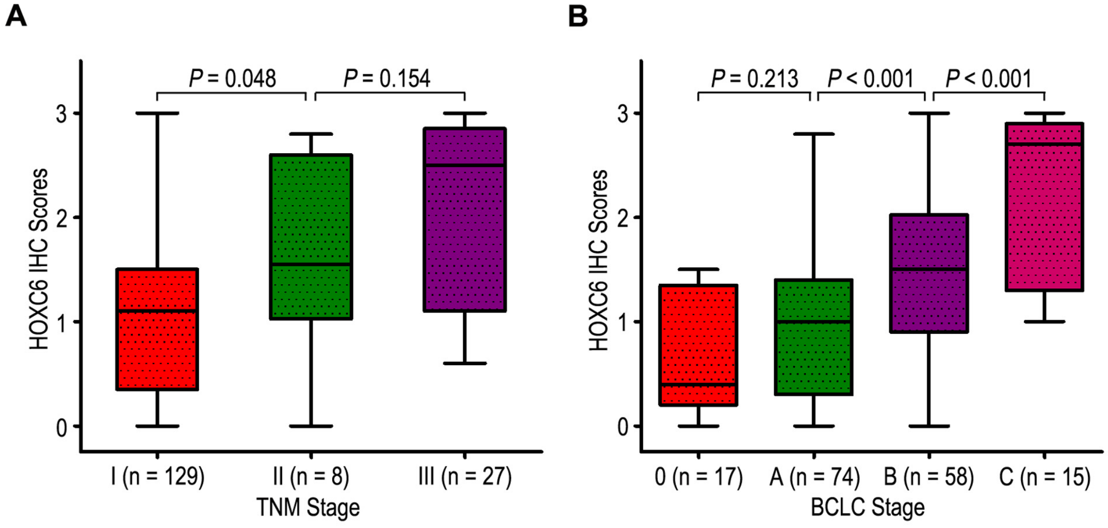

Figure S1. Comparsion of HOXC6 expression by TNM stage and BCLC stage. HOXC6 expression is gradually increased as tumors progress in TNM stage (A) and BCLC stage (B). Statistical analysis was performed by Student's *t*-test.
